# Activation of platelet protease-activated receptor-1 induces epithelial-mesenchymal transition and chemotaxis of colon cancer cell line SW620

**DOI:** 10.3892/or.2015.3897

**Published:** 2015-04-03

**Authors:** YITAO JIA, SUQIAO ZHANG, LINGLING MIAO, JINGBAO WANG, ZUJIAN JIN, BIN GU, ZHIHUI DUAN, ZHAOLONG ZHAO, SHUNMAO MA, WENJIN ZHANG, ZHONGXIN LI

**Affiliations:** 1 Department of Oncology, Hebei General Hospital, Shijiazhuang, Hebei 050051, P.R. China; 2 Second Department of Surgery, The Fourth Hospital of Hebei Medical University, Shijiazhuang, Hebei 050011, P.R. China; 3 Second Department of Surgery The Bethune International Peace Hospital, Shijiazhuang, Hebei 050082, P.R. China; 4 Department of Gynecology and Obstetrics, The Yiwu Affiliated Hospital of Zhejiang University, Yiwu, Zhejiang 322000, P.R. China; 5 Department of Surgery, Hebei Medical University Affiliated North China Petroleum Bureau General Hospital, Renqiu, Hebei 062552, P.R. China; 6 Centre of Breast Cancer, The Fourth Hospital of Hebei Medical University, Shijiazhuang, Hebei 050011, P.R. China

**Keywords:** epithelial-mesenchymal transition, chemotaxis, platelet, protease activated receptor-1, transforming growth factor β1, colorectal cancer

## Abstract

The aim of the present study was to examine the role of protease-activated receptor-1 (PAR1)-stimulated platelet activation in the epithelial-mesenchymal transition (EMT) and migration of colon cancer cells, and to identify the underlying mechanisms. TFLLR-NH_2_, a PAR1 agonist, was used to activate platelets and the platelet supernatants were used to treat the SW620 colon cancer cell line. Expression of E-cadherin and vimentin on SW620 cells was detected by immunofluorescence and western blotting, and the level of the transforming growth factor β1 (TGF-β1) was measured using ELISA following the activation of platelets by TFLLR-NH_2_. miR-200b expression was detected using quantitative PCR in SW620 cells. In order to investigate the chemotactic ability of the SW620 cells, the expression of CXC chemokine receptor type 4 (CXCR4) was measured by flow cytometry. Transwell migration assays were performed following exposure of the cells to the supernatant of PAR1-activated platelets. SW620 cells cultured in the supernatant of TFLLR-NH_2_-activated platelets upregulated E-cadherin expression and downregulated the vimentin expression. In the in vitro platelet culture system, a TFLLR-NH_2_ dose-dependent increase of secreted TGF-β1 was detected in the supernatant. The activation of PAR1 on the platelets led to the inhibition of miR-200b expression in the SW620 cells that were cultured in platelet-conditioned media. The number of SW620 cells that penetrated through the Transwell membrane increased with the dose of TFLLR-NH_2_ used to treat the platelets. The percentage of CXCR4-positive SW620 cells was significantly higher when they were exposed to the supernatant of platelets cultured for 24 h with PAR1 agonist than when cultured in non-conditioned media (40.89±6.74 vs. 3.47±1.40%, P<0.01). Platelet activation with a PAR1 agonist triggered TGF-β secretion, which induced EMT of SW620 human colon cancer cells via the downregulation of miR-200b expression, and activated platelets had a chemotactic effect on colon cancer cells mediated by the upregulation of CXCR4 on the cell surface.

## Introduction

Regional or distal lymph-node metastasis is the major cause of mortality for patients with malignant tumors. Although tumor metastasis has been extensively studied, the exact mechanisms remain to be determined. Epithelial-mesenchymal transition (EMT) is a process whereby epithelial cells transform morphologically and phenotypically into fibroblasts or mesenchymal cells, and gain migratory ability (1). EMT is considered to be the first step of tumor metastasis (2,3). During EMT, epithelial cells lose their polarity and cell-cell adhesion, develop cyto-skeletal changes and obtain increased migratory potential and mobility (4). This process is concurrent with the alteration of various molecular markers, including downregulation of the epithelial markers E-cadherin and β-catenin, and upregulation of the mesenchymal phenotypic markers vimentin and N-cadherin (5). EMT has been shown to be closely associated with the invasion and metastasis of various malignancies, including gastric, colorectal, breast, liver and ovarian cancer (6–10). Numerous factors and processes are involved in the EMT of cancer cells, such as transforming growth factor (TGF)-β, hepatocyte growth factor (HGF), c-Met amplification, epidermal growth factor receptor (EGFR) mutation, and transcription factors ZEB1/2, Snail, Twist and Tiam1 (3). Among these, TGF-β is considered one of the most important inducers of EMT. TGF-β exerts an antitumor activity through the suppression of tumor growth at the early stage of tumor development, while at the late stage, it promotes tumor metastasis and EMT of tumor cells by reducing the adhesion molecule expression, accelerating tumor neovascularization and activating proteases that promote tumor metastasis (11).

MicroRNAs (miRNAs) are small non-coding RNA molecules (containing ~22 nucleotides) that are widely found in eukaryotes and viruses and regulate various cell functions. The mature miRNA binds to the non-coding region at the 3′-terminus of the target mRNA to regulate gene expression post-transcriptionally. miRNAs are also closely associated with tumor development and progression (12). The miR-200 family, an important class of EMT mediators, is found to mediate the proliferation, migration, invasion and metastasis of epithelial cell-derived malignant tumor cells in response to transforming growth factor β1 (TGF-β1) by regulating the expression of ZEB1/2 (13). In addition, to maintain the integrity of intestinal epithelium, TGF-β1 induces EMT by suppressing Smad2 in a miR-200b-dependent manner (14).

Tumor metastasis is known to be organ specific. as tumor cells undergo EMT and become migratory, they may develop directional migration via chemokines. Chemokines are preferentially expressed in organs or tissues, and along with cell surface proteins, including integrins, they regulate the homing of multiple blood cell subsets to specific anatomic sites. During tumor development, the stromal cell-derived factor 1 (SDF-1)/CXC chemokine receptor type 4 (CXCR4) axis has been shown to be involved in the metastasis of various types of human cancer (15,16).

The role of platelets in tumor progression has attracted increasing attention. Platelets, a major component of blood, contain α and dense granules and lysosomes, and play an important role in hemostasis, thrombosis and inflammation (17). In addition, platelets promote tumor metastasis. An increase in the platelet count is positively correlated with tumor metastasis, while a reduction in platelet count or inhibition of platelet function markedly inhibits tumor metastasis (18–21). The platelet α granules contain many substances, such as vascular endothelial growth factor (vEGF), SDF-1, platelet-derived growth factor (PDGF) and TGF-β, which are involved in tumor progression when excreted (22,23). Activation of the platelet α granule is mediated by the proteinase-activated receptor (PAR) signaling pathway (23,24). PAR is a subfamily of the seven-pass transmembrane G-protein-coupled receptors, including PAR1, PAR2, PAR3 and PAR4 (25). PAR1 is expressed on platelets and is activated by thrombin or tissue factor in the tumor microenvironment (26,27). Notably, PAR1 is also expressed in multiple tumor cells, and inhibition of PAR1 may suppress tumor growth and metastasis (28).

In clinical practice, thrombocythemia is predominantly found in patients with advanced tumors. It was generally thought that thrombocythemia develops only at the late stages of cancer, and platelets are only involved in the growth and metastasis of advanced tumors (29). However, it has been demonstrated that a large number of tumor cells migrate into the bloodstream even at an early stage, and these circulating tumor cells (CTCs) constitute nidi for tumor metastasis and recurrence (30). EMT and chemotaxis are essential for CTCs to enter the vasculature. In addition, platelet activation has been found to occur in the early stages of tumor development (30). Previous findings have shown that only direct contact between platelets and breast cancer cells induces EMT of breast cancer cells via the TGF-β signaling pathway, suggesting that platelets induce EMT of tumor cells thus facilitating their entry into the bloodstream (31). Since tumor cells develop EMT prior to entering the bloodstream, we speculate that platelets may not need to directly contact tumor cells to induce their EMT, and may instead have a chemotactic effect on tumor cells. The aim of the present study was to investigate whether PAR1-activated platelets induce a colon cancer cell line to undergo EMT without direct contact with cancer cells, and to understand the role of platelets in the early-phase metastasis of colon cancer cells.

## Materials and methods

### Cell lines

The SW620 human colon cancer cell line was purchased from the Cell Bank of the Type Culture Collection of the Chinese Academy of Sciences (Shanghai, China). The cells were cultured in McCoy’s 5A medium containing 10% fetal bovine serum (FBS), 1% penicillin and 1% streptomycin at 37°C in 5% CO_2_.

### Antibodies and reagents

The PAR1 agonist TFLLR-NH_2_ was obtained from Sigma-Aldrich (St. Louis, MO, USA). The human CD62P-FITC antibody was purchased from AK-4 (eBioscience, inc., San Diego, CA, USA). E-cadherin and vimentin rabbit anti-human antibodies were purchased from Abcam (Cambridge, MA, USA). PathScan^®^ EMT Duplex IF kit was purchased from Cell Signaling Technology, Inc. (Shanghai, China). The ELISA kit for TGF-β1 was obtained from Neobioscience Technology Co., Ltd. (Beijing, China). Transwell chambers (Corning Inc., Lowell, MA, USA) were used for the EMT assays. The cells were stained for flow cytometry using PE anti-human CD184/CXCR4 (12G5; BioLegend, San Diego, CA, USA).

### Preparation of the platelets

Whole blood samples were collected from 10 healthy volunteers (8 men and 2 women, none of whom had any medical history or had received anticoagulants one week prior to blood sample collection). The blood samples were centrifuged at 200 x g for 10 min to yield three cell layers: platelet rich plasma (PRP) on the upper layer, white cells at the middle layer, and red cells at the lower layer. Approximately 2/3 of the PRP layer were transferred to a centrifugation tube containing 50 ng/ml prostaglandin E1 (PGE1), centrifuged at 22°C for 10 min and the supernatant was removed. The sediment platelets were washed twice with 5 ml of phosphate-buffered saline (PBS) without calcium or magnesium. The platelets were re-suspended in solutions containing 5 mM glucose and 0.25% bovine serum albumin (BSA), and adjusted to a concentration of 150,000/*μ*l for the subsequent experiments.

### Test for the optimal dose of platelet PAR1 agonist

To investigate platelet activation following treatment with various concentrations of TFLLR-NH_2_, CD62P (P-selectin) was used as a marker of platelet activation, and the rate of CD62P-positive platelets was detected using flow cytometry. TFLLR-NH_2_ was added to the platelet culture at final concentrations of 1, 3, 5, 7 and 9 *μ*M in McCoy’s 5A medium containing 10% FBS, and incubated at 37°C for 15 min, while non-treated platelets served as controls. The CD62P expression in the platelets was determined using flow cytometry (FC500 flow cytometer; Beckman Coulter, Inc., Chaska, MN, USA).

### Detection of EMT

Based on the results of the above tests, 3 *μ*M TFLLR-NH_2_ was selected for subsequent experiments to determine whether activation of platelet PAR-1 induces EMT of the SW620 colon cancer cell line. Three groups were assigned: i) group 1, 100 *μ*l of activated platelet supernatant + SW620 cells, ii) group 2, 100 *μ*l of supernatant of untreated platelets + SW620 cells and iii) group 3, 100 *μ*l of medium + SW620 cells. Three independent experiments were conducted.

After 24 h of culture at 37°C, the SW620 cells were stained for E-cadherin and vimentin using the PathScan^®^ EMT Duplex IF kit, and their expression was observed under a four-channel CLS-4HS laser confocal microscope (Thorlabs Inc., Newton, NJ, USA). Three independent experiments were conducted.

After incubation in 6-well plates for 24 h, the E-cadherin and vimentin protein expression in the SW620 cells was detected using western blotting. Briefly, the cultured cells were washed once with PBS buffer and lysed in lysis buffer (1% SDS, 50 mM Tris, pH 7.4, 0.15 M NaCl, 1 mM NaF, 10 mM phenylmethylsulfonyl fluoride, 1 mM sodium orthovanadate, 1 mM EDTA) for 5 min and passed through a 27-gauge needle. Lysates were centrifuged at 12,000 x g for 1 min, the supernatants were collected, and protein concentrations were determined using a Bio-Rad DC protein assay (Bio-Rad, Hercules, CA, USA). Equal amounts of protein were separated by 10% SDS-PAGE and transferred to nitrocellulose membranes. The membranes were blocked with 3% BSA in TBST (10 mM Tris, pH 7.5, 150 mM NaCl, 0.1% Tween-20) for 1 h. Primary antibodies (1:200) and secondary antibodies were used according to the manufacturer’s instructions. The blots were analyzed and quantified with the MCID imaging software (Imaging Research Inc., St. Catharines, ON, Canada). β-actin served as an internal reference.

### Transwell migration assay

We assessed whether activation of platelet PAR-1 induces chemotaxis of the SW620 colon cancer cell line using a Transwell migration assay. Transwell chambers with an 8-*μ*m pore diameter and a 6.5 mm membrane diameter were used in a chemotaxis assay. Three groups were assigned: i) activated platelet supernatant group (cells were incubated in 600 *μ*l of the supernatant of the platelets activated by TFLLR-NH_2_ at a final concentration of 1, 3, 5, 7 or 9 *μ*M in McCoy’s 5A medium containing 10% FBS), ii) non-activated platelet supernatant group (same as above but without TFLLR-NH_2_ treatment), and iii) blank control group (cells were treated with McCoy’s 5A medium containing 10% FBS).

The upper Transwell chamber was filled with SW620 cells at a concentration of 2.5×10^5^/100 *μ*l, and the chemotaxis chamber was incubated at 37°C in 5% CO_2_ for 18 h. The nuclei of the cells migrating through the Transwell membrane were stained with 5 *μ*g/ml of DAPI for 10 min, and its fluorescence signal was observed under an inverted fluorescence microscope. The number of cells at five randomly selected fields was counted, and the mean count was calculated. Each experiment was repeated three times.

### Quantification of TGF-β1 levels in the platelet supernatant by ELISA

The supernatants of the platelets treated with TFLLR-NH_2_ at a final concentration of 1, 3, 5, 7 or 9 *μ*M in McCoy’s 5A medium containing 10% FBS and PBS were collected, and the TGF-β1 released from platelets α granules was detected using an ELISA kit according to the manufacturer’s instructions.

### Quantitative PCR of miR-200b expression

Quantitative PCR (qPCR) was performed to detect the effect of the activated platelet supernatant on miR-200b expression in the SW620 cells. Platelets from healthy donors were incubated in 3 *μ*M TFLLR-NH_2_ in McCoy’s 5A medium containing 10% FBS for 15 min, and the platelet supernatants were collected. The SW620 cells were treated with 0, 60, 120, 240 or 480 *μ*l of the supernatant for 24 or 48 h. Total RNA was isolated from the cells using TRIzol^®^ reagent, and its integrity was checked on agarose gel. After reverse transcription, qPCR was performed using a miR-200b primer (part no.: 4427975; assay ID: 002251) based on the specific cDNA template in 20 *μ*l of the reaction system containing 10 *μ*l of TaqMan^®^ Universal PCR Master Mix, 1 *μ*l of diluted PreAmp product, 1 *μ*l of microRNA assay (all from ABI, Santa Monica, CA, USA) and 8 *μ*l of nuclease-free water under the following conditions: at 95°C for 10 min, followed by 40 cycles at 95°C for 15 sec, and at 60°C for 60 sec. U6 snRNA (part no.: 4427975; assay ID: 001973; ABI) served as an internal reference. The relative miR-200b expression was estimated using the 2^−ΔΔCt^ method.

### Flow cytometric detection of CXCR4 expression on the SW620 cell surface

The TFLLR-NH_2_ and non-activated platelet groups were assigned. SW620 cells were seeded in 6-well plates at a concentration of 1.0×10^6^ cells/well. The platelet supernatant (100 *μ*l) activated at a final concentration of 3 *μ*M TFLLR-NH_2_ in McCoy’s 5A medium containing 10% FBS was added to the TFLLR-NH_2_ group, while the same volume of medium was transferred to the blank control. After a 24 h culture, the cells were collected, digested with pancreatic enzymes and the cell density was adjusted to 1.5×10^7^ cells/ml. The CXCR4 level on the SW620 cell surface was determined using flow cytometry. Experiments were repeated three times.

### Statistical analysis

Data are presented as mean ± standard deviation (SD). Statistical analyses were performed using the statistical software SPSS version 13.0 (SPSS, Inc., Chicago, IL, USA). The statistical significance of the differences between the means was determined by the Student’s t-test when analyzing two independent samples, while analysis of variance (ANOVA) was employed for multiple independent samples. Rank sum test was used to make comparisons among multiple groups when the variances of the groups were heterogeneous. P<0.05 was considered to indicate a statistically significant result.

## Results

### Activation of platelets by the PAR agonist TFLLR-NH_2_

**Figure 1:**
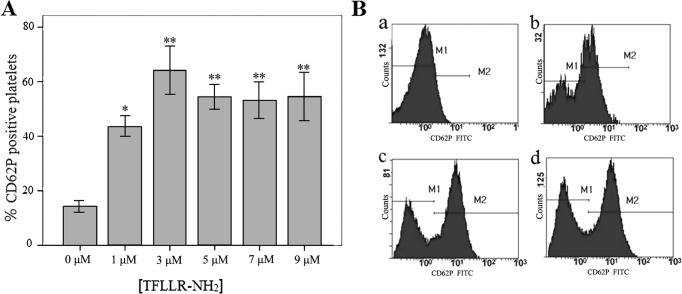
Flow cytometry detection of CD62P in the platelets treated with the PAR1 agonist TFLLR-NH_2_. (a) CD62P expression in the platelets treated with different concentrations of TFLLR-NH_2_ as detected by flow cytometry (mean ± standard deviation of three independent experiments). *P<0.05 and **P<0.01. (B) Representative FACS plots for CD62P expression in the platelets activated by (a) 0, (b) 1, (c) 3 and (d) 10 *μ*M TFLLR-NH_2_. M1, CD62P-negative region; M2, CD62P-positive region. PAR1, protease-activated receptor-1.

To detect platelet activation following treatment with various concentrations of TFLLR-NH_2_, the rate of CD62P (P-selectin)-positive platelets was determined using flow cytometry. The rates of CD62P-positive platelets were (43.52±1.5)%, (64.22±3.60)%, (54.46±1.82)%, (53.15±2.7)% and (54.63±3.66)% in the platelets treated with TFLLR-NH_2_ at a final concentration of 1, 3, 5, 7 and 9 *μ*M, respectively, all of which were significantly greater (P<0.05) than the rates in the blank control (no TFLLR-NH_2_) group (14.35±0.86)% (Fig. 1A and B). Thus, 3 *μ*M was selected as the dose of TFLLR-NH_2_ for platelet activation.

### EMT of SW620 cells

**Figure 2:**
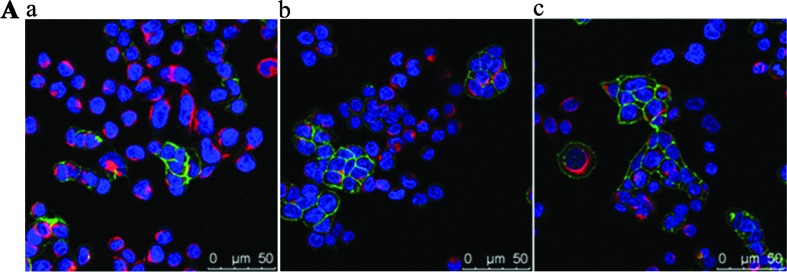

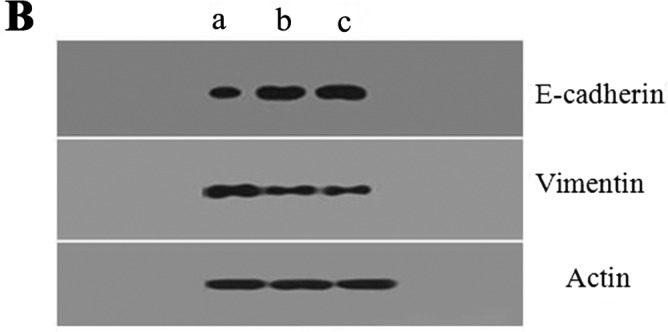
(A) Confocal immunofluorescence images showing the expression of vimentin (red) and E-cadherin (green) in the SW620 cells after 24 h treatment with (a) PAR1-activated platelet supernatant, (b) non-activated platelet supernatant and (c) no treatment. SP magnification, ×400. (B) Expression of E-cadherin or vimentin protein in the SW620 cells detected by western blotting. (a) PAR1-activated platelets supernatant, (b) non-activated platelets supernatant and (c) medium control. PAR1, protease-activated receptor-1.

To detect whether platelet activation induced EMT of the SW620 cells, immunofluorescence and western blotting were employed to determine the expression of EMT markers E-cadherin and vimentin. Laser confocal microscopy showed that the supernatant of TFLLR-NH_2_-activated platelets triggered the downregulation of E-cadherin expression and the upregulation of vimentin expression (Fig. 2A) in the SW620 cells compared to the cells treated with medium or with the supernatant of untreated platelets. Western blot analysis showed a similar finding (Fig. 2B). The grey value of E-cadherin was 0.225 for cells treated with activated platelet supernatant, which was lower than that for the cells treated with medium or with the supernatant of untreated platelets (0.758 and 0.769, respectively). However, the grey value of vimentin was 0.616 for cells treated with activated platelet supernatant, which was greater than that for the cells treated with medium or with the supernatant of untreated platelets (0.211 and 0.234, respectively). These findings suggested that platelet activation may induce the EMT of the SW620 cells.

### The supernatant of TFLLR-NH_2_-activated platelets induces SW620 cell migration

**Figure 3:**
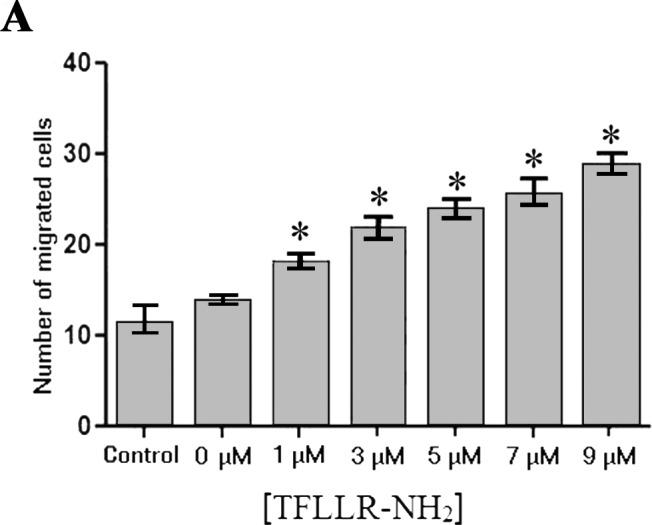

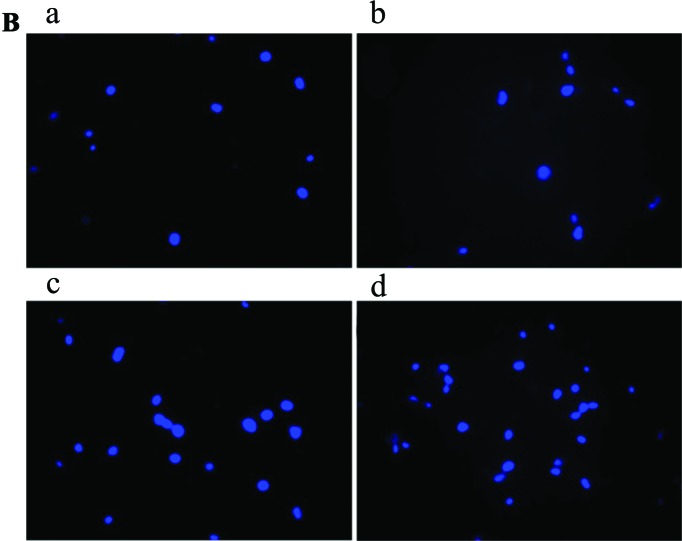
(A) Migration of the SW620 cells induced by platelet activation examined by Transwell migration assay. The number of the migrated SW620 cells induced by platelet supernatants extracted from the platelets activated by different concentrations of TFLLR-NH_2_. *P<0.05 compared to the control. (B) Fluorescence microscopy image of the upper Transwell chamber after migration of the SW620 cells towards the activated platelet supernatant. The nuclei were stained with DAPI. (a) Media only control, (b) no TFLLR-NH_2_ in the platelet culture, (c) 1 *μ*M TFLLR-NH_2_ and (d) 7 *μ*M TFLLR-NH_2_.

A chemotaxis assay was performed to investigate the chemotactic effect of platelet activation on tumor cells. Our findings showed no significant difference in the number of SW620 cells that migrated through the Transwell membrane/field between the group with the supernatant of untreated platelets and the blank control group (11.33±2.08 vs. 13.67±0.58, P>0.05). When using conditioned media from the platelets treated with TFLLR-NH_2_ at a final concentration of 1, 3, 5, 7 and 9 *μ*M, the number of SW620 cells migrating through the membrane to the platelet supernatant was 18±1, 21.67±1.53, 23.67±1.53, 25.33±2.08 and 28.67±1.53, respectively (Fig. 3A), all of which were significantly greater (P<0.05) than the numbers in the blank control (no TFLLR-NH_2_) group and in the group with the supernatant of the untreated platelets (P<0.05). A dose-dependent increase in the number of the cells migrating through the Transwell membrane was observed (Fig. 3B). These findings indicated that the supernatant of the TFLLR-NH_2_-activated platelets had a chemotactic effect on the SW620 cells.

### TGF-β1 levels in the TFLLR-NH_2_-activated platelet supernatant

**Figure 4:**
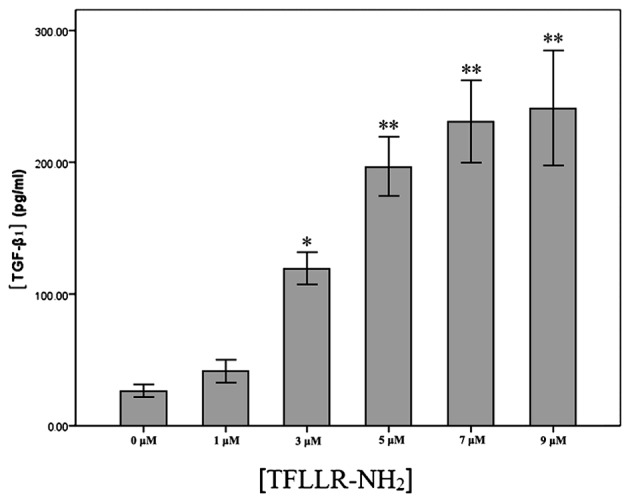
The levels of TGF-β1 released by platelets treated with different concentrations of TFLLR-NH_2_ as detected by ELISA. The TGF-β1 secretion was significantly higher in the activated platelet group than that in the control, and the TGF-β1 level increased in a dose-dependent manner in the activated platelet groups. *P<0.05 and **P<0.01 vs. the control group. TGF-β1, transforming growth factor β1.

The TGF-β1 levels were 41.42±3.60, 119.27±5.16, 197.11±9.21, 231.37±7.26 and 241.33±17.60 pg/ml in the supernatants of the platelets activated by TFLLR-NH_2_ at a final concentration of 1, 3, 5, 7 and 9 *μ*M, respectively, reflecting a dose-dependent increase in the TGF-β1 secretion, and the TGF-β1 level was significantly higher in the activated platelet supernatant than in that of the control group (26.31±1.96) (Fig. 4).

### miR-200b expression in SW620 cells

**Figure 5:**
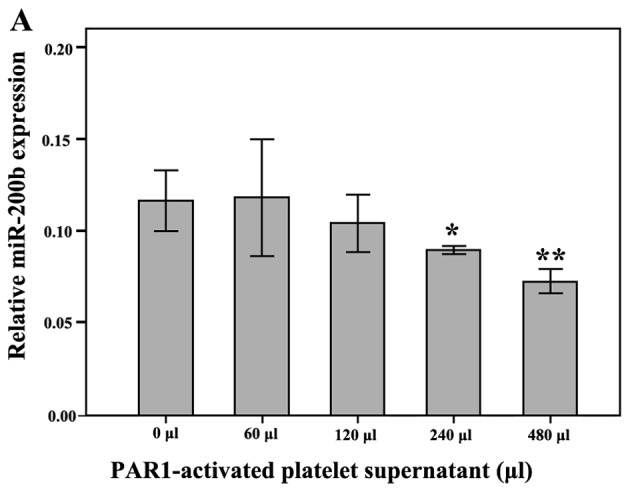

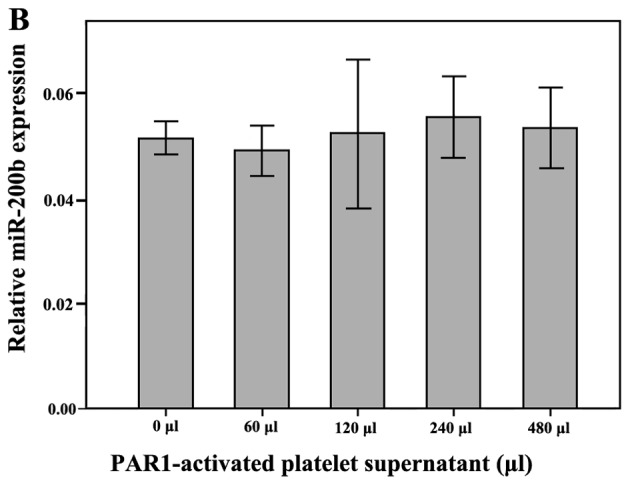
Expression of miR-200b of the SW620 treated with different volumes of the PAR1-activated platelet supernatant detected by qPCR at (A) 24 or (B) 48 h. (a) after 24 h the groups treated with 60 and 120 *μ*l of platelet supernatant did not indicate significant difference (P>0.05) compared to the control group, whereas those treated with 240 and 480 *μ*l showed a significantly lower miR-200b expression. *P<0.05 and **P<0.01. PAR1, protease-activated receptor-1.

Although miR-200b has been shown to be involved in the regulation of EMT (14), the effect of the platelet activation on miR-200b expression remains unclear. qPCR was performed to investigate the effect of 3 *μ*M TFLLR-NH_2_-treated platelet supernatant on the miR-200b expression in the SW620 cells. Our findings showed that following treatment with activated platelet supernatant for 48 h, no significant difference was found in the relative miR-200b expression between the non-treated platelet supernatant control and the TFLLR-NH_2_ treatment group (P>0.05) (Fig. 5B). The relative miR-200b expression was 0.118±0.013, 0.105±0.006, 0.090±0.001 and 0.073±0.003 in SW620 cells treated with 60, 120, 240 and 480 *μ*l of the platelet supernatant for 24 h, respectively, compared to 0.117±0.007 in the blank control group. However, the relative miR-200b expression was lower in the SW620 cells treated with 240 and 480 *μ*l of the platelet supernatant than in the control group (Fig. 5A, P<0.05), suggesting that the miR-200b expression appeared to decline with increasing volumes of the platelet supernatant, while in the SW620 cells treated with 60 and 120 *μ*l of the platelet supernatant there were no significant differences with the non-treated platelet supernatant control group (P>0.05).

### Effect of TFLLR-NH_2_-activated platelet supernatant on CXCR4 expression in the SW620 cells

**Figure 6:**
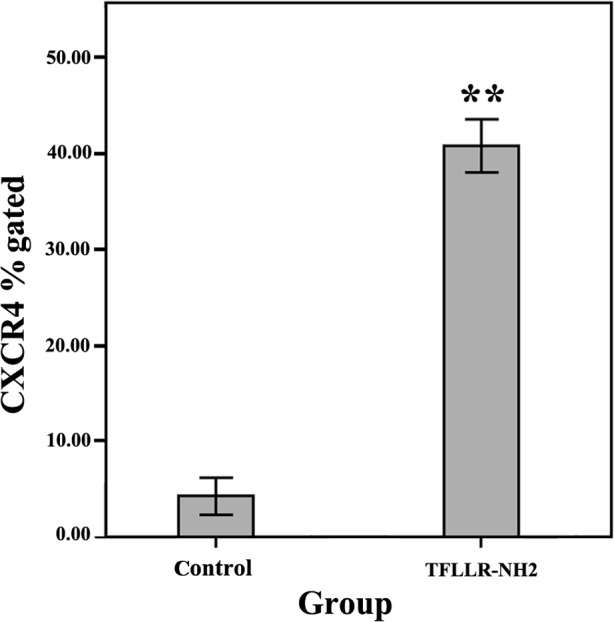
CXCR4 expression of SW620 cells treated with culture medium (control) or with supernatants of the platelets cultured in 3 *μ*M TFLLR-NH_2_ as detected by flow cytometry. **P<0.01. CXCR4, CXC chemokine receptor type 4.

Cell surface CXCR4, when bound to its ligand SDF-1, induces the directional migration of tumor cells (32). The aforementioned results showed that TFLLR-NH_2_-activated platelets, not only induced the chemotaxis of the SW620 cells, but also stimulated TGF-β1 secretion by platelets. Flow cytometry was used to investigate the effect of platelet activation on CXCR4 expression on SW620 cells. Our findings showed that (40.89±6.74)% of the SW620 cells were positive for CXCR4 24 h after treatment with the supernatant of the platelets activated by 3 *μ*M TFLLR-NH_2_, which was significantly greater than that (3.47±1.40)% in the non-activated platelet group (P<0.01; Fig. 6).

## Discussion

In the present study, we found that PAR1 agonist TFLLR-NH_2_-activated platelets released TGF-β1 leading to the upregulation of CXCR4 and the inhibition of miR-200b expression in SW620 cells, ultimately inducing EMT-phenotype and migration in vitro. Our findings suggest that the activation of platelet PAR1 may be important in the initial stages of tumor metastasis, and early antiplatelet therapy may be of great significance to suppress colorectal cancer metastasis and improve the survival rate.

Platelet-tumor cell interaction is extremely complex in tumor microenvironments. It has been reported that tumor cells upregulate the expression of tissue factor (TF) leading to an increase in thrombin, which activates platelet PAR1 or PAR4 (33,34). In addition, tumor cells can directly secrete thrombin into microenvironments to activate platelets (35). inflammation and angiogenesis are usually present inside tumors, and the distribution of new blood vessels is disorganized. These pathological changes may cause the slowdown of blood circulation in and surrounding the tumors, thereby increasing the probability of tumor-activating platelet deposition (36), which may promote tumor cell growth and metastasis.

Labelle *et al* (37) found that the platelet-breast cancer cell interaction promoted tumor cell EMT and metastasis. Since the supernatant of the thrombin-activated platelets did not induce tumor cell EMT within 48 h, the direct contact between platelets and tumor cells was considered essential for the development of EMT (38). However, this does not concur with our findings. in the present study, the supernatant of platelets activated by a PAR1 agonist TFLLR-NH_2_, was found to induce EMT of the SW620 cells within 24 h, indicating that tumor cell EMT may occur without the direct contact between platelets and tumor cells. In addition, Labelle et al (37) reported that the induction of breast cancer EMT by platelets required at least one week. However, the sensitivity to cytokines may vary in tumor cells, resulting in varying potentials and speeds of EMT. For example, it has been shown that EMT occurs in the tumor cells NMuMG, A549 and MDA-MB-231 24 h after the addition of TGF-β (38).

Our findings show that the activated platelets secreted TGF-β1, and conditioned media from the activated platelet culture led to the downregulation of miR-200b expression in the SW620 cells. Accumulating evidence has demonstrated that multiple factors released by platelets induce tumor cell EMT, of which TGF-β is the most extensively investigated (37,38). TGF-β has been found to promote the metastasis of multiple tumors through the induction of cancer cell EMT (12,14,39). Besides activating the Smad pathway, TGF-β induces tumor cell EMT through other means, including the Ras-Erk/MAPK, p38/MAPK, JNK, Rho GTPase and PI3K/Akt pathways (12). In addition, TGF-β has been reported to mediate EMT via the regulation of miRNA transcription (14). It has been shown that TGF-β1 signaling downregulates the expression of the miR-200 family by activating Akt2, leading to the upregulation of ZEB1 and ZEB2 (39). Moreover, ZEB2 binds to the miR-200 promoter to inhibit its transcription, resulting in the formation of a negative feedback loop, which further stimulates the EMT of tumor cells (40).

In addition, results of the present study have shown that PAR1 agonist-activated platelets had a chemotactic effect on the SW620 colon cancer cell line. It is well known that activated platelets may be involved in the chemotaxis of various cells, and thus participate in various pathological processes, such as inflammation, thrombogenesis and arteriosclerosis (41). During inflammation, platelets promote their adhesion to other cells in the blood by expressing receptors such as β3 integrins, and are involved in the chemotaxis of inflammatory cells by releasing a large number of chemokines (42). Our findings confirm the chemotactic effect of platelets on malignant tumor cells. If a solid tumor exceeds 2 mm in diameter, new blood vessels form, a process accompanied by the infiltration of inflammatory cells. When platelets pass through tumors via the bloodstream, they may be activated by specific cytokines released by tumor cells or by mesenchymal cells in tumor microenvironments, and activated platelets may in turn induce tumor cell EMT and migration into blood vessels. Therefore, some clinically diagnosed early-stage tumors are virtually at an ‘advanced phase’. This may explain the reason for some cancer patients developing early-stage metastasis. It has been shown that an antiplatelet agent such as aspirin may reduce the metastatic rate of malignant tumors and improve prognosis (43).

In addition, our findings show that the activated platelet culture supernatant upregulated the expression of CXCR4 on the surface of SW620 cells, suggesting platelets actively induce the migration of tumor cells into blood vessels. The chemokine receptor CXCR4 is widely present in multiple tumors, and a high CXCR4 expression is a predictive marker of tumor metastasis and results in a worse prognosis (44–46). For example, a low CXCR4 expression is detected in normal breast tissues, while a high expression is found in breast cancer tissues (47). The CXCR4-SDF-1 interaction may lead to actin polymerization and pseudopod formation, thereby resulting in chemotaxis and invasion (47), while the specific blockade of CXCR4 inhibits the metastasis of breast cancer cells to lymph nodes and bone marrow (47). In addition, a high CXCR4 expression was found to correlate with bone metastasis in prostate cancer, while a neutralizing antibody against CXCR4 may block the bone metastasis of prostate cancer (48).

The interaction between tumor and microenvironment is complex, and the interaction between tumor and the cell components in the microenvironment is potent. The present study only investigated the effect of static platelets on tumor cells. Future studies are required to evaluate the impact of platelets on tumor cells in dynamic bloodstream. In addition to TGF-β1, platelet-derived PDGF plays an important role in inducing cell EMT (49). Notably, platelets have recently been found to enter tumor tissues in animal models (50). Further studies are warranted to evaluate the role of platelets in tumor progression and the difference between intratumoral and intra-vascular platelet-mediated tumor activation.

In conclusion, PAR1-activated platelets may induce EMT of the SW620 colon cancer cell line via the TGF-β pathway, and they provide chemotactic signals to the SW620 cells which lead to the upregulation of CXCR4 on the cancer cell surface. The present study provides evidence showing a molecular mechanism potentially underlying colorectal cancer metastasis and contributes to supporting the potential value of early antiplatelet therapy for colorectal cancer.
